# Novel combinatorial therapy of oncolytic adenovirus AdV5/3-D24-ICOSL-CD40L with anti PD-1 exhibits enhanced anti-cancer efficacy through promotion of intratumoral T-cell infiltration and modulation of tumour microenvironment in mesothelioma mouse model

**DOI:** 10.3389/fonc.2023.1259314

**Published:** 2023-11-20

**Authors:** Mariangela Garofalo, Magdalena Wieczorek, Ines Anders, Monika Staniszewska, Michal Lazniewski, Marta Prygiel, Aleksandra Anna Zasada, Teresa Szczepińska, Dariusz Plewczynski, Stefano Salmaso, Paolo Caliceti, Vincenzo Cerullo, Ramon Alemany, Beate Rinner, Katarzyna Pancer, Lukasz Kuryk

**Affiliations:** ^1^Department of Pharmaceutical and Pharmacological Sciences, University of Padova, Padova, Italy; ^2^Department of Virology, National Institute of Public Health, National Institute of Hygiene (NIH) - National Research Institute, Warsaw, Poland; ^3^Division of Biomedical Research, Medical University of Graz, Graz, Austria; ^4^Centre for Advanced Materials and Technologies, Warsaw University of Technology, Warsaw, Poland; ^5^Department of Bacteriology and Biocontamination Control, National Institute of Public Health, National Institute of Hygiene (NIH) - National Research Institute, Warsaw, Poland; ^6^Departament of Sera and Vaccines Evaluation, National Institute of Public Health, National Institute of Hygiene (NIH) - National Research Institute, Warsaw, Poland; ^7^Laboratory of Bioinformatics and Computational Genomics, Faculty of Mathematics and Information Science, Warsaw University of Technology, Warsaw, Poland; ^8^Laboratory of Functional and Structural Genomics, Centre of New Technologies, University of Warsaw, Warsaw, Poland; ^9^Drug Research Program (DRP), ImmunoViroTherapy Lab (IVT), Division of Pharmaceutical Biosciences, Faculty of Pharmacy, University of Helsinki, Helsinki, Finland; ^10^Helsinki Institute of Life Science (HiLIFE), University of Helsinki, Helsinki, Finland; ^11^Translational Immunology Program (TRIMM), Faculty of Medicine Helsinki University, University of Helsinki, Helsinki, Finland; ^12^Digital Precision Cancer Medicine Flagship (iCAN), University of Helsinki, Helsinki, Finland; ^13^Department of Molecular Medicine and Medical Biotechnology and CEINGE, Naples University Federico II, Naples, Italy; ^14^Oncobell Program of Bellvitge Biomedical Research Institute (IDIBELL), ProCure Program of Catalan Institute of Oncology (ICO), Avinguda de la Granvia de l’Hospitalet, L'Hospitalet de Llobrega, Barcelona, Spain; ^15^Clinical Science, Valo Therapeutics, Helsinki, Finland

**Keywords:** immune checkpoint inhibitors, immunotherapy, oncolytic adenovirus, mesothelioma, anti PD-1, TILs, CD40L, ICOSL

## Abstract

**Introduction:**

Malignant mesothelioma is a rare and aggressive form of cancer. Despite improvements in cancer treatment, there are still no curative treatment modalities for advanced stage of the malignancy. The aim of this study was to evaluate the anti-tumor efficacy of a novel combinatorial therapy combining AdV5/3-D24-ICOSL-CD40L, an oncolytic vector, with an anti-PD-1 monoclonal antibody.

**Methods:**

The efficacy of the vector was confirmed *in vitro* in three mesothelioma cell lines – H226, Mero-82, and MSTO-211H, and subsequently the antineoplastic properties in combination with anti-PD-1 was evaluated in xenograft H226 mesothelioma BALB/c and humanized NSG mouse models.

**Results and discussion:**

Anticancer efficacy was attributed to reduced tumour volume and increased infiltration of tumour infiltrating lymphocytes, including activated cytotoxic T-cells (GrB+CD8+). Additionally, a correlation between tumour volume and activated CD8+ tumour infiltrating lymphocytes was observed. These findings were confirmed by transcriptomic analysis carried out on resected human tumour tissue, which also revealed upregulation of CD83 and CRTAM, as well as several chemokines (CXCL3, CXCL9, CXCL11) in the tumour microenvironment. Furthermore, according to observations, the combinatorial therapy had the strongest effect on reducing mesothelin and MUC16 levels. Gene set enrichment analysis suggested that the combinatorial therapy induced changes to the expression of genes belonging to the “adaptive immune response” gene ontology category. Combinatorial therapy with oncolytic adenovirus with checkpoint inhibitors may improve anticancer efficacy and survival by targeted cancer cell destruction and triggering of immunogenic cell death. Obtained results support further assessment of the AdV5/3-D24-ICOSL-CD40L in combination with checkpoint inhibitors as a novel therapeutic perspective for mesothelioma treatment.

## Introduction

Malignant mesothelioma (MM) is an aggressive and very rare type of cancer that develops within the layer of mesothelial cells. The worldwide incidence of this malignancy has risen over the last decade, and an increase in the number of cases in the future is anticipated. Unfortunately, MM is almost universally lethal, and the median survival time from diagnosis is up to 12 months. Although new treatment options are currently available, they are not curative, and new drugs are highly needed to provide hope for mesothelioma patients (1–3).

The immune system plays a pervasive role in the prevention and treatment of cancer. Malignant tumors, on the other hand, can evolve a variety of immune suppression strategies (4). Several immunomodulating drugs have been explored as anti-cancer treatments and launched into clinical settings in recent years. Among them immune checkpoint inhibitors (CPIs), acting against PD-1 (programmed cell death protein 1), PD-L1 (programmed death ligand 1), and CTLA-4 (cytotoxic T-lymphocyte-associated protein 4), have exhibited antitumor activity in a variety of tumor types, including metastatic melanoma, lung cancer, and breast malignancies (5). These CPIs have demonstrated significant therapeutic efficacy in metastatic carcinomas by reverting effector T-cell depletion and malfunction, improving anti-tumoral characteristics, and therefore enhancing T-cell activation (6). However, results of clinical trials show only limited overall survival of patients treated with anti-PD-1. Nevertheless, clinical trials PROMISE-Meso (NCT02991482) and CONFIRM (NCT03063450) suggest that PD-1 inhibitors, like pembrolizumab and nivolumab, have modest but clinically relevant activity in relapsed MM (7).

A promising anti-cancer strategy in solid cancer therapy is virotherapy. Oncolytic viruses (OVs) can infect and reproduce specifically within tumor cells, inevitably culminating to tumor cell lysis (8–13). OVs can elicit powerful, systemic, and persistent anti-tumor immunity in addition to direct and localized anti-tumor action (14–19). Many molecules are released by dying tumor cells, triggering antitumor immunity, and generating therapeutic responses even at distant tumor locations (20–23). Nevertheless, despite extensive research, oncolytic viruses have shown limited efficacy against solid tumors as monotherapy. Therefore, the refinement of novel and more efficacious oncolytic vectors is needed.

Immunotherapy functions well for metastatic carcinomas and can complement standard chemotherapeutics and radiotherapy (24). It has been shown that combining OVs with CPIs can elicit a synergistic antitumor efficacy that may contribute to improved therapeutic outcomes (14, 20, 25, 26). Thus, the present study was designed to evaluate the anti-tumor effectiveness of the combinatorial therapy encasing oncolytic vector AdV5/3-D24-ICOSL-CD40L, expressing two powerful co-stimulatory molecules: inducible co-stimulator ligand (ICOSL) and CD40 ligand (CD40Land CD154) (25), with an anti-PD-1 monoclonal antibody, in both immunodeficient and humanized xenografted mesothelioma H266 mouse models. Importantly, it was previously shown that intratumoral (i.t.) therapy with oncolytic adenovirus armed with ICOSL can activate innate immunity and upregulates the expression of T-cell co-stimulatory receptors (23). In addition, it has been reported elsewhere that an OV coding for CD40L induced tumor regression *in vivo* by demonstrating apoptotic impacts, leading to an increased calreticulin (CRT) exposure and HMGB1 (high mobility group box 1) and ATP (adenosine triphosphate) output (27).

Moreover, we studied possible correlation between the level of tumor-infiltrating lymphocytes (TILs) and anti-cancer effect (tumor volume control). Importantly, transcriptomic analyses have been carried out to better understand clinical responses to therapy and seek for prognostic markers. Our study demonstrated that AdV5/3-D24-ICOSL-CD40L co-administered with human anti-PD-1 offers anti-cancer benefits in tested advanced mesothelioma mouse models. Profiling of the tumor microenvironment (TME) revealed sustained AdV5/3-D24-ICOSL-CD40L-induced immune cell infiltration correlating with tumor growth inhibition. Together, these results support further assessment of the virus in combination with anti-PD-1 for the management and treatment of malignant mesothelioma patients.

## Materials and methods

### Cell lines, viruses, anti-PD-1 antibodies

MSTO-211H (ACC 390, DSMZ, Germany) and NCI-H226 (H226, CRL-5826, ATCC, Manassas, VA) human malignant biphasic mesothelioma cells were grown in RPMI 1640 supplemented with 10% heat inactivated fetal bovine serum (FBS) (Gibco Laboratories), 2 mM L-glutamine (Gibco Laboratories), and 1% penicillin and streptomycin (Gibco Laboratories). Mero-82 (09100105-1VL) was a human epithelioid mesothelioma cell line procured from Sigma Aldrich and grown in Hams F10 with 15% heat-inactivated FBS (Gibco Laboratories), 2 mM L-glutamine (Gibco Laboratories), and 1% penicillin and streptomycin (Gibco Laboratories). Cell Bank Australia provided the mouse mesothelioma cell line AB12. The murine cell line was grown in RPMI 1640 medium containing 1% penicillin/streptomycin (Gibco Laboratories), 2 mM L-glutamine (Gibco Laboratories), and 10% FBS (Gibco Laboratories). The AdV5/3-D24-ICOSL-CD40L (consisting of a 24-bp deletion in E1A Conserved Region 2 (CR2), a CMV-ICOSL-CD40L expression cassette inserted in the E3 region, and Ad5/3 hybrid fiber), and AdV5/3-D24 (consisting of a 24-bp deletion in E1A Conserved Region 2 (CR2), and Ad5/3 hybrid fiber) adenovirus vectors utilized in this study are chimeric type 5/3 adenoviruses created and amplified using viral production procedures (25). Anti-mouse CD279 (PD1) antibody was purified and resuspended as per the manufacturer’s instructions (BioLegend). Anti-PD-1 antibodies (pembrolizumab) were purchased from Merck.

### CAR, CD46, DSG2, and PD-L1 expression in cancer cell lines

H226, MSTO-211H, and Mero-82 were stained with mouse monoclonal anti-CAR antibody (Santa Cruz Biotech, Dallas, TX, USA) followed by 1:2,000 Alexa-Fluor 488 secondary antibody (Abcam, Cambridge, UK) or mouse monoclonal anti-DSG2 antibody (Abcam, Cambridge, UK) and then with 1:2,000 Alexa-Fluor 488 secondary antibody (Abcam, Cambridge, UK). Rabbit anti-PD-L1 antibodies (Alexa Fluor 488, Abcam, ab209959) were used to measure PD-L1 expression (at least 1×10^4^ cells/events were examined by flow cytometry, BD FACSCantoTM II (Franklin Lakes, NJ, USA)). Flow cytometry analysis was performed on FlowJo v10 software.

### Cell viability: MTS cytotoxicity assay

H226, MSTO-211H, and Mero-82 mesothelioma cell lines were seeded at a density of 1×10^4^ cells/well in a 96-well plate and kept under standard growth conditions (RPMI 1640/Hams F10, supplemented with 5% FBS, 1% L-glutamine, and 1% penicillin/streptomycin). After overnight incubation, cells were treated as follows: (i) culture media, (ii) AdV5/3-D24-ICOSL-CD40L (0.1, 1, 10, and 100 viral particles (VP)/cell), and (iii) AdV5/3-D24 (0.1, 1, 10, 100 VP/cell), with or without anti-PD-1 (100 µg/mL). The viability of the cells was assessed 96 h after treatment employing the CellTiter 96 AQueous One Solution Cell Proliferation Assay (MTS) as directed by the manufacturer (Promega, Madison, WI, USA). A 96-well plate spectrophotometer (Victor NivoTM, PerkinElmer) was used to detect the absorbance at 490 nm. The experiment was run in triplicate.

### Immunogenic cell death

*CRT exposure*. At 5×10^5^ cells per well, cell lines were seeded in triplicate into six-well plates. According to the treatment combinations listed above, cells were infected with 100 VP/cell of the tested oncolytic adenovirus and/or anti-PD-1 drugs (50 μg/mL). After 48 h, cells were collected and stained with 1:1,000 diluted rabbit polyclonal anti-calreticulin antibody (Abcam, Cambridge, UK) for 40 min at 4°C, followed by flow cytometry (Franklin Lakes, NJ, USA) (14).

*HMGB-1 release*. Cell lines were seeded in triplicate into 96-well plates at a density of 1×10^4^ cells/well and infected with 100 VP/cell of evaluated oncolytic adenovirus and/or anti-PD-1 drugs as per the treatment combinations shown above. Supernatants were collected after a time span of 72 h, and HMGB-1 was quantified using an ELISA kit according to the manufacturer’s instructions (MBL International, Woburn, MA) (14).

*ATP release*. Cell lines were seeded in triplicates onto 96-well plates at a density of 1×10^4^ cells/well and treated as described before. Following 72 h, supernatants were recovered, and luminometric analysis was performed using the ATP Determination Kit (Promega, Madison, WI) according to the manufacturer’s procedure (Varioscan Flash, Thermo Fisher Scientific, Waltham, MA) (14).

### *In vivo* studies

Animal procedures were approved by the Austrian Federal Ministry of Science and Research, the Italian Ministry of Health, and the Warsaw University of Life Sciences’ II Local Ethical Committee for Animal Experiments. Mesothelioma xenografts were established by injecting 6 × 10^6^ H226 cells subcutaneously (s.c.) into one or both sides of BALB/c nude mice (n=5 mice per group, 5 tumors per group) or human CD34+ hematopoietic stem-cell-engrafted NSG variant mice (hu-CD34+, Jax Laboratories) (n=4 mice per group, 8 tumors per group). During the acclimation and treatment phase, all animals were monitored for clinical symptoms, morbidity, and death daily. Clinical signs in animal health scoring have been monitored (**Supplementary Table S1**). Selected organs such as the spleen, liver, heart, kidneys, lungs, brain, and tumors underwent basic necropsy assessment. Prior to the start of treatment, tumors of sizes ~5 × 5 mm in diameter were randomized. Mice were given treatments according to the ones enlisted in **Supplementary Table S2**, **S3** (immunodeficient mesothelioma H226 mouse model, humanized mesothelioma H226 mouse model). At least twice a week, the size of the tumor was measured using a caliper in two dimensions. At each timepoint, the longest and shortest diameters of the tumor were measured, and the tumor volume was computed using the formula 0.52 length × (width)^2^. To monitor the tumor development of H226 cells in BALBc nude mice and NSG variant mice (hu-CD34+), micro-ultrasound measurement (Vevo3100, Fujifilm VisualSonics) was performed at least twice a week. For ultrasound examinations, the animals were anesthetized using 2% isoflurane and 2.5 L/min O_2_ and then placed on a heated platform. The region of interest was depilated, and the tumor investigated and measured in coronal and transverse planes using transducers of 52–70 MHz. After ultrasound investigation, the animals were cleaned from gel residues, transferred back to their home cage, and monitored until fully awake.

### Immune cell infiltrates—*ex vivo* analyses

The percentage number of human immune cell populations were monitored by flow cytometry: human CD45+ lymphocytes (BD, cat. number: 564105), T cells hCD3+ (BD, cat. number: 555339), CD4+ T cells (hCD3+ hCD4+, BD, cat. number: 557852), CD8+ T cells (hCD3+ hCD8+, BD, cat. number: 560179), activated CD8+ (hCD3+ hGrB+ hCD8+, BD, cat. number: 560212), and FoxP3 (hCD3+ hCD4+ hFoXP3+, BD, cat. number: 560046). Tumors were harvested and subsequently dissociated with cell strainer (day 35—end of study). Immune cells were isolated by following the protocol described earlier (28). After dissociation, cells were washed using BD Perm/Wash™ buffer (cat. no. 554723) and stained with antibodies for 30 min at 4°C in the dark and then suspended in stain buffer FBS (BD, cat no. 554656). Samples were acquired using BD Lyric FACS Flow. The populations were gated with forward and side scattering (FSC-A/SSC-A dot plot) in leukocytic regions. Flow cytometry analysis was performed on FlowJo v10 software.

### Quantitative real-time PCR

The quantification of adenoviral DNA copies has been performed according to the protocol described earlier (1). Samples were analyzed using LighCycler qPCR machine (LighCycler 480, Roche, Basel, Switzerland).

### Gene expression analyses

Whole transcriptome analysis, using total RNA sequencing (Illumina NextSeq, sequenced in paired-end mode) of available tumors (end of study) from humanized H226 mice was performed. Eight publicly available RNA-seq data for H226 cell line (GSM4117346) (29) were used to enrich the control group that originally comprised of two samples. In brief, reads were first trimmed of nucleotides from both ends if their quality in Phred scale was below 30; afterwards, only reads longer than 40 bp were kept. Reads were subsequently aligned to the human genome (hg38) using the align function from the Rsubread package (ver. 2.10.1). Only reads with the flags 99, 147, 83, or 163 were kept for further analysis. Reads were assigned to genes, as defined by the GENCODE annotation (ver. 39), using the feature counts function. A total of 18,254 genes for which at least seven samples had at least four reads assigned were kept. As data come from two experiments, batch effects were removed with the ComBat function from the SVA package (ver. 3.44) using non-parametric adjustments. Differential expression between groups (control, 10 samples; AdV5/3-D24-ICOSL-CD40L, six samples; pembrolizumab, three samples; AdV5/3-D24-ICOSL-CD40L + pembrolizumab, two samples) was performed using limma (ver. 3.52). Gene ontology (GO) enrichment was analyzed with topGO (ver. 2.48) with the Fisher’s exact test and weight01 algorithm. Relative levels of different immune cells were calculated from RNA-seq data using the quanTIseq and MCP-counter methods (30) using the immunedeconv package (ver 2.0.4) (31).

### Statistical analysis

*In vitro* and *in vivo* variables were analyzed using GraphPad Prism software (version 9). A repeated measures with ANOVA and Mann–Whitney t-test were used in the statistical analysis. The Pearson correlation coefficient was utilized to look for possible correlations between tumor volume and the percentage of CD4+, CD8+, GrB+CD8+, and FoxP3 cells in tumor-infiltrating lymphocytes.

## Results

### Evaluation of cell viability by MTS cytotoxicity assay

**Figure 1:**
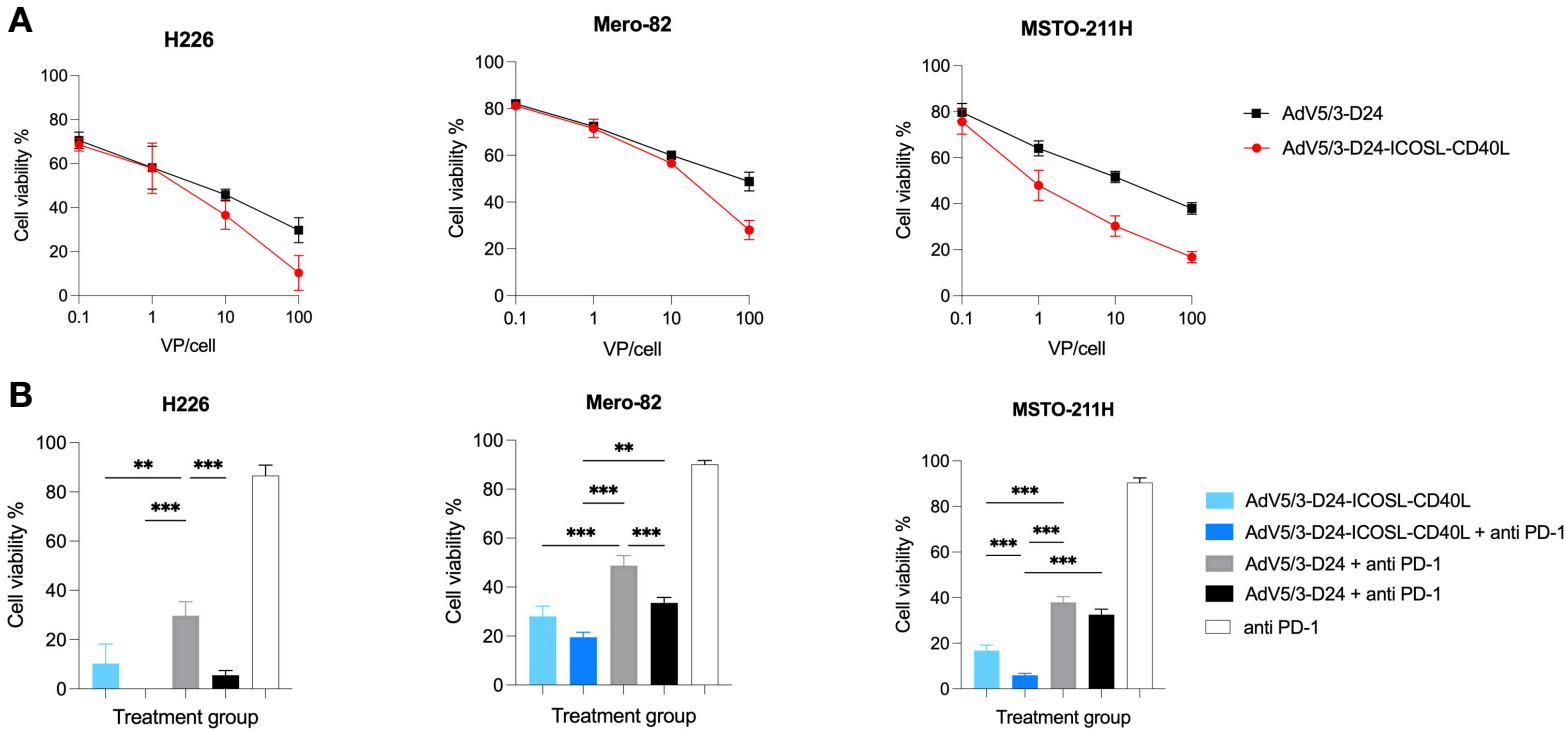
*In vitro* cytotoxicity assay (MTS assay). **(A)** AdV5/3-D24-ICOSL-CD40L and AdV5/3-D24 at concentrations of 0.1, 1, and 10, and 100VP/cell were used to assess cell viability 96-h after infection. **(B)** Combinatory treatment with AdV5/3-D24-ICOSL-CD40L and AdV5/3-D24 at concentrations 100VP/cell with or without anti-PD-1 (100 µg/mL) were used to assess cell viability 96 h after infection. Statistical analyses were carried out with Mann–Whitney t-test. Error bars, mean ± SEM; **p ≤ 0.01, ***p<0.001.

The *in vitro* cytotoxicity efficacy of AdV5/3-D24-ICOSL-CD40L was tested in H226, Mero-82, and MSTO-211H cell lines to check whether the presence of the double transgenes into the viral backbone could affect the *in vitro* oncolytic activity. Oncolytic potency of tested oncolytic adenoviruses was confirmed. Treatment with oncolytic adenovirus AdV5/3-D24-ICOSL-CD40L showed enhanced *in vitro* efficacy compared to AdV5/3-D24 in all tested mesothelioma cell lines. Although not statistically significant (**Figure 1A**), the results suggest that the incorporation of co-stimulator transgenes (ICOSL and CD40L) did not impair oncolytic properties of the vector. Interestingly, the combinatory therapy of both tested oncolytic adenoviruses with anti-PD-1 showed enhanced killing efficacy *in vitro* on three tested cell lines (**Figure 1B**) presumably due to drugs toxicity.

### Immunogenic cell death assessment

Markers for immunogenic cell death (ICD), such as the exposure of calreticulin to cell surface and the extracellular release of ATP and HMGB1 (32), were measured from mesothelioma cell cultures after exposure to the virus, anti-PD-1, or combination of both. The infected cell lines with tested oncolytic adenoviruses resulted in ICD *in vitro* (expression of calreticulin, release of ATP, and HM-GB1). Immunogenic cell death was observed when treated with the virus and combinatory therapy (**Supplementary Figure S1**). H226 cell line was the most susceptible for cell death when treated with the virus and with the combinatory therapy.

### CAR, CD46, DSG2, and PD-L1 expression in mesothelioma cell lines

All mesothelioma cells lines (MSTO-211H, H226, and Mero-82) expressed high level of CD46 (93%, 99%, and 99%, respectively) and DSG2 (99%, 99%, and 98%, respectively) on their surfaces. Finally, MSTO-211H (88%), H226 (48%), and Mero-82 (94%) expressed CAR. All mesothelioma cell lines express PD-L1 on cell surface (>98%) (**Supplementary Figure S2**).

### *In vivo* efficacy study in immunodeficient xenograft mesothelioma H226 mouse model

**Figure 2:**
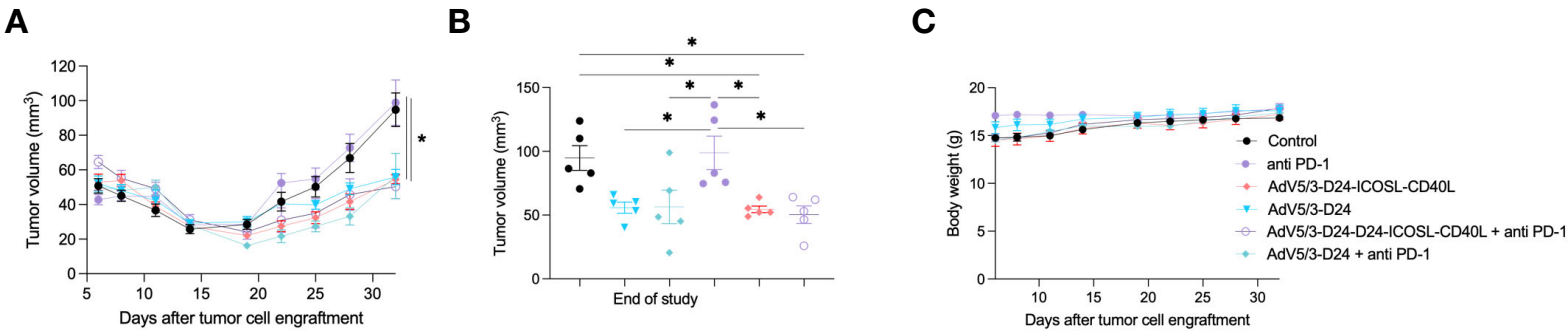
Antitumor efficacy of AdV5/3-D24, AdV5/3-D24-ICOSL-CD40L, and anti-PD-1 and the combinatory therapy in mesothelioma H226 xenograft immunodeficient BALB/c nude model (5×10^6^ cells/flank, n=5 per group). **(A)** Prior to the start of treatment, tumors of sizes ~5 × 5 mm in diameter were randomized. Once tumors have been formed, the treatment has been initiated. Mice received 1×10^8^ VP viruses i.t., 200 μg anti-PD-1 i.v. on days 0, 3, 6, 9, 12, and 15. The control group received PBS administered in a same scheme as treated groups. Tumor volume (mm^3^) was measured through the study. At the conclusion of the study, mice were sacrificed, and tumors were extracted. **(B)** Tumor volume (mm^3^) measured at the end of the study (day 32). **(C)** Body weight was measured throughout the study. Statistical analyses were carried out with ANOVA test. Error bars, mean ± SEM; *p ≤ 0.05.

Next, we carried out experiment in immunodeficient xenograft mesothelioma H226 mouse model, where we aimed at assessing oncolytic properties of the virus. Due to known limitations of the model, such as lack of a thymus, impaired immune system, immunological properties of the vector, anti-PD-1 were not able to be properly assessed. Anti-cancer efficacy was observed in mice treated with oncolytic adenovirus Ad5/3-D24-ICOSL-CD40L (vs. mock, p ≤ 0.05), AdV5/3-D24, combination therapy (AdV5/3-D24 with anti-PD-1 vs. mock, p ≤ 0.05), and when mice received Ad5/3-D24-ICOSL-CD40L + anti-PD-1. No statistically significant difference in anti-cancer efficacy has been observed between tested oncolytic adenoviruses (Ad5/3-D24-ICOSL-CD40L vs. AdV5/3-D24) (**Figures 2A, B**). As expected, no treatment efficacy was observed in mice treated with anti-PD-1 alone (**Figures 2A, B**). At the end of the study, the average volume size of a tumor for mice treated with the AdV5/3-D24, Ad5/3-D24-ICOSL-CD40L, and in their combinatory therapy was, respectively, 56, 55, and 56, 50 mm^3^ compared to 99 mm^3^ (anti-PD-1) and 95 mm^3^ (control). The treatment was well tolerated (**Figure 2C**). All mice survived till the end of study (day 32). Adenoviral DNA copy number assessed by qPCR revealed presence of adenoviral DNA in tumor cells (**Supplementary Figure S3**).

### *In vivo* efficacy and immunomodulatory properties of tested agents in humanized xenograft mesothelioma H226 mouse model

**Figure 3:**
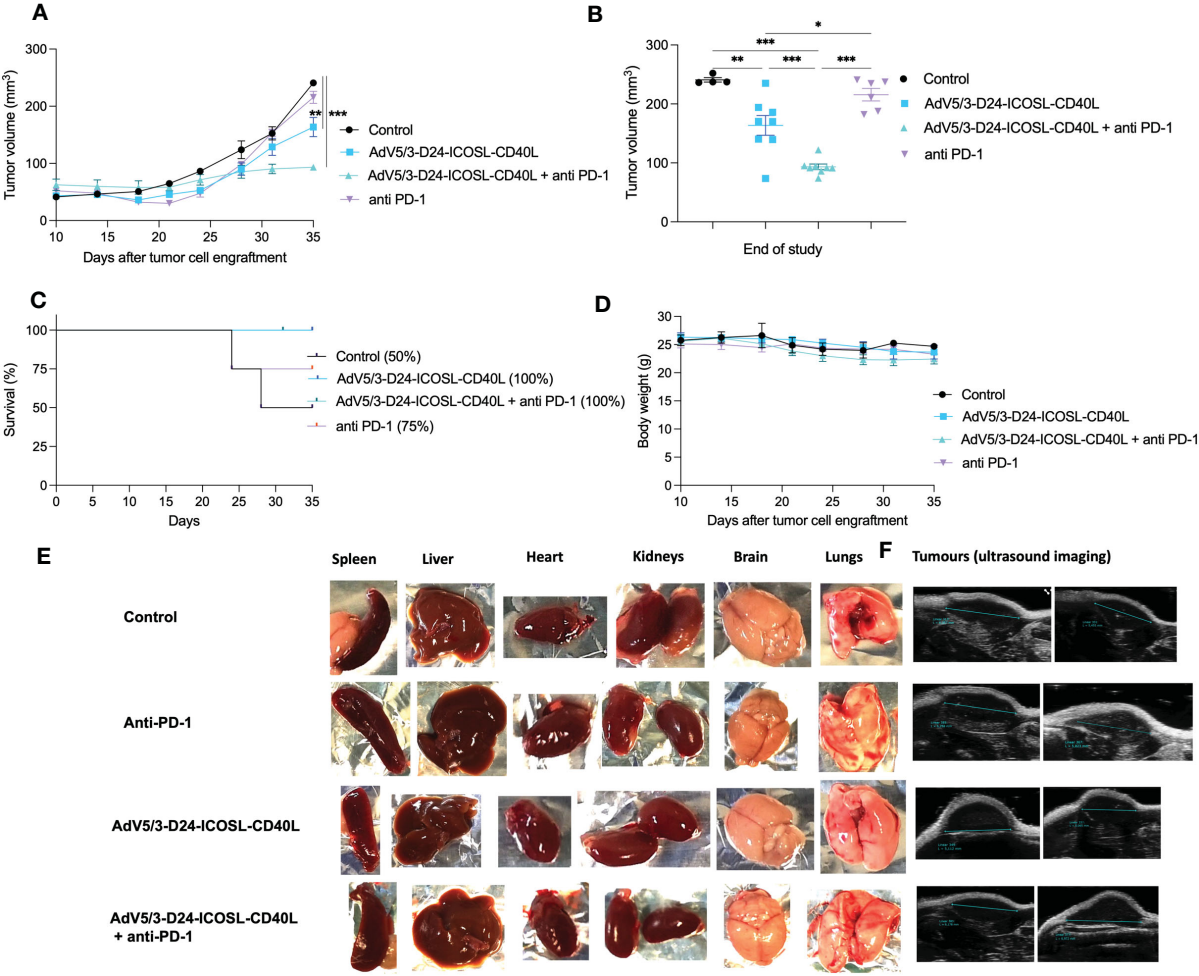
Antitumor efficacy of AdV5/3-D24-ICOSL-CD40L anti-PD-1 and the combinatory therapy in humanized mesothelioma H226 NSG nude model (5×10^6^ cells/flank, n=4 mice per group (8 tumors per group). **(A)** Prior to the start of treatment, tumors of sizes ~5 × 5 mm in diameter were randomized. Once tumors have been formed, the treatment has been initiated. Mice received 2×10^9^ VP viruses i.t., 200 μg anti-PD-1 i.v. on days 0, 3, 6, 9, 12, and 15. The control group received PBS administered in a same scheme as treated groups. Tumor volume (mm^3^) was measured through the study. At the conclusion of the study, mice were sacrificed, and tumors were extracted to determine tumor volume. **(B)** Tumor volume (mm^3^) measured at the end of the study (day 35). **(C)** Survival profile was calculated by Kaplan–Meier test. **(D)** Body weight was measured throughout the study. **(E)** Morphological examination of organs collected from mice in control and treated with the virus plus anti-PD-1 group. **(F)** Representative ultrasound images for each group. Statistical analyses were carried out with ANOVA test. Error bars, mean ± SEM; *p ≤ 0.05, **p ≤ 0.01, ***p<0.001.

**Figure 4:**
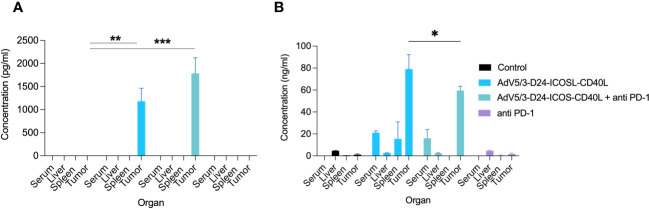
Evaluation of ICOSL and CD40L expression in various mouse organs after the treatment with oncolytic adenoviruses, anti-PD-1, and their combinations in humanized mesothelioma H226 model. **(A)** ICOSL concentration was measured from mouse organs (liver, tumor, and spleen) and blood collected at sacrifice after the treatment with ELISA kit (RayBiotech, ELH-B7H2-1) according to manufacturer’s instructions. **(B)** CD40L was detected from mouse organs (liver, tumor, and spleen) and blood collected at sacrifice after the treatment with ELISA kit (RayBiotech, ELH-CD40L-1) as per the instructions laid down by the manufacturer. Statistical analyses were carried out with ANOVA test. Error bars, mean ± SM; *p ≤ 0.05, **p ≤ 0.01, ***p<0.001.

**Figure 5:**
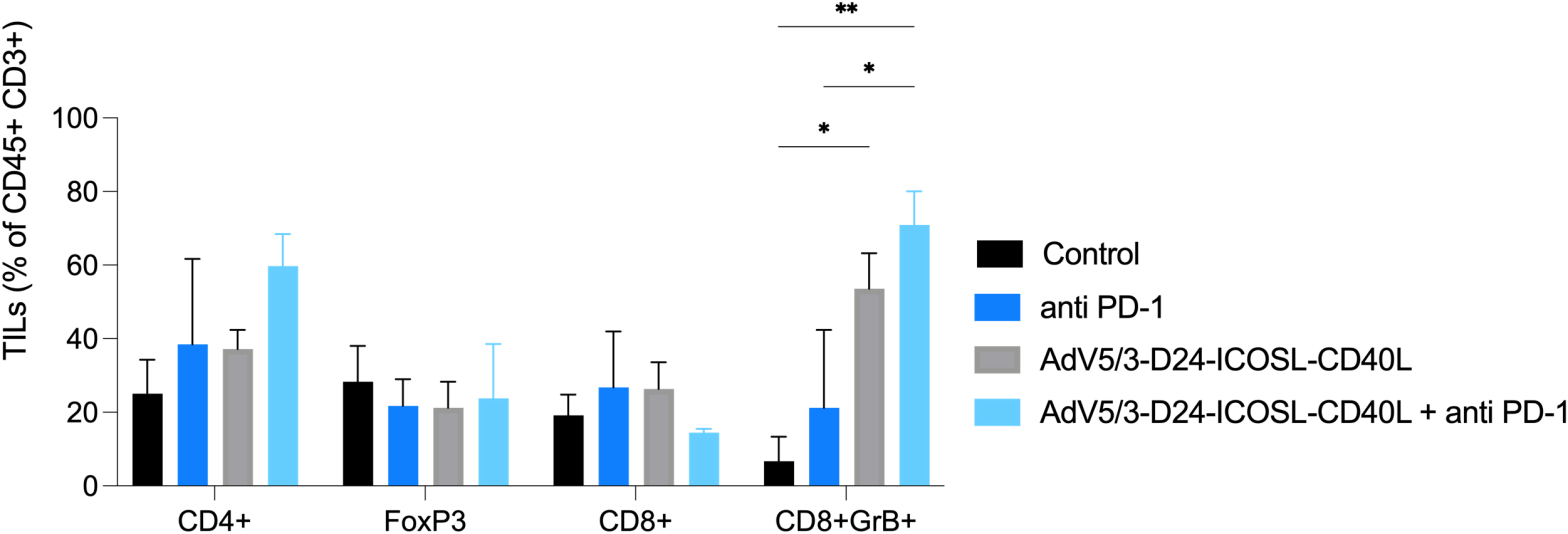
Levels of tumor infiltrating lymphocytes CD4+, CD8+, FoxP3, and GrB+CD8+ expression in collected tumors (end of study). Samples were acquired using BD Lyric FACS Flow. Statistical analyses were carried out with ANOVA test. Error bars, mean ± SEM; *p ≤ 0.05, **p ≤ 0.01.

**Figure 6:**
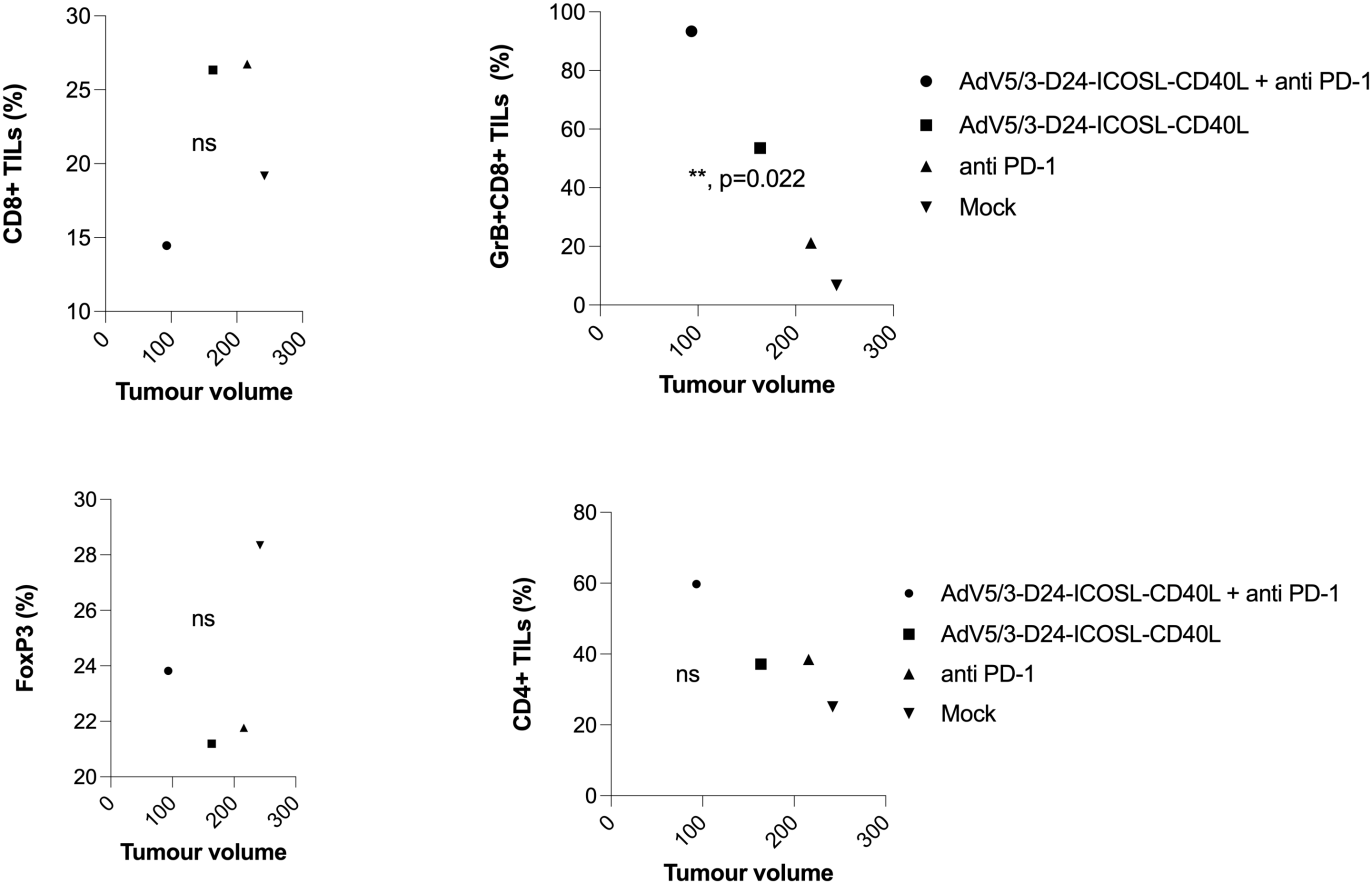
Immunomodulatory properties of tested agents in humanized xenograft mesothelioma H226 NSG nude model. At the end of the study, mice were euthanized and tumors collected for immunological analyses from four groups: (i) control, (ii) AdV5/3-D24-ICOSL-CD40L, (iii) anti-PD-1, and (iv) the combinatory therapy (end of study). Tumor-infiltrating lymphocytes CD4+, CD8+, FoxP3, and CD8+GrB+ mean expression has been assessed in collected tumors. Samples were acquired using BD Lyric FACS Flow. Statistical analyses were carried out with ANOVA test; ns, not significant. Error bars, mean ± SEM.

To study oncolytic properties of the vector and immunostimulatory functions of the tested agents, humanized xenograft mesothelioma H226 mouse model was exploited. Improved anti-cancer efficacy was observed in combination therapy with AdV5/3-D24-ICOSL-CD40L plus anti-PD-1 versus mock (p ≤ 0.01) and in mice treated with AdV5/3-D24-ICOSL-CD40L (vs. mock, p ≤ 0.05) (**Figure 3A**). After 35 days of treatment, the average volume size of a tumor in combination regime group was 93 mm^3^ compared to 216 mm^3^ (anti-PD-1), 164 mm^3^ (virus alone), 241 mm^3^ (control) (**Figure 3B**). Survival of 100% was reported for mice treated with the virus alone and combinatory therapy (**Figure 3C**). The treatment was well tolerated (**Figure 3D**), no pathological changes in necropsy assessment were found (**Figures 3E, F**). *Ex vivo* analyses revealed local production of ICOSL and CD40L transgenes encoded by the AdV5/3-D24-ICOSL-CD40L (**Figure 4**) and presence of adenoviral DNA copies in the tumors (**Supplementary Figure S3**). Enhanced infiltration of activated cytotoxic (GrB+ CD8+ T cells) tumor-infiltrating T cells has been reported in mice treated with the combination regimen (**Figure 5**, p ≤ 0.01, combination therapy vs. control). Statistically significant correlation between tumor volume and GrB+CD8+ TILs was seen (**Figure 6**, p=0,022).

### Gene expression analyses

**Figure 7:**
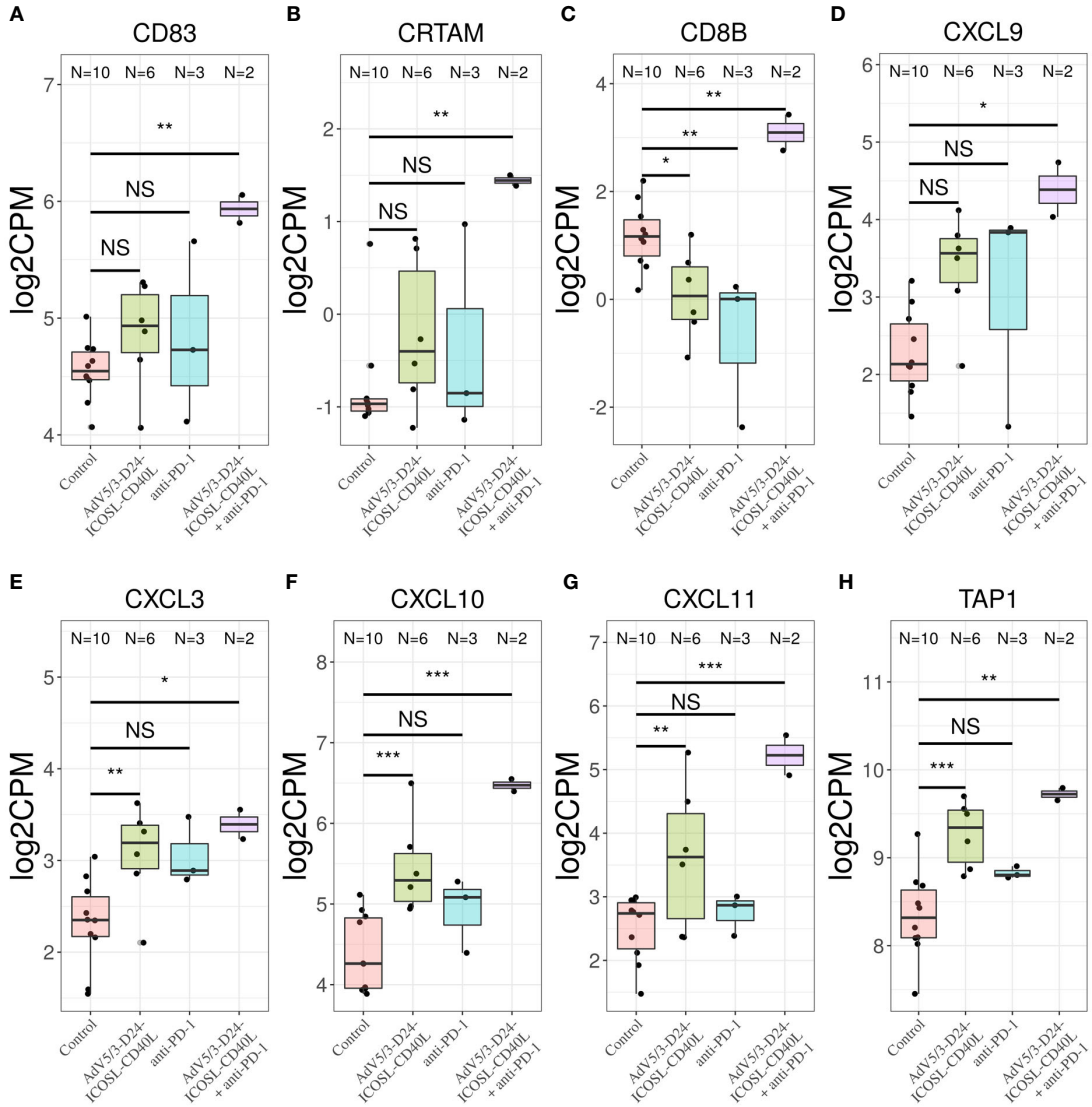
Transcriptomic analysis revealed upregulation of **(A)** CD83, **(B)** CRTAM, **(C)** CD8B, and **(D)** CXCL9 only in samples treated with combinatorial therapy. Transcriptomic analysis revealed upregulation of chemokines **(E)** CXCL3, **(F)** CXCL10, **(G)** CXCL11, and **(H)** TAP1 in samples treated with only AdV5/3-D24-ICOSL-CD40L, only anti-PD-1, or both AdV5/3-D24-ICOSL-CD40L + anti-PD-1 regimen, *p ≤ 0.1, **p ≤ 0.05, ***p<0.01; ns, not significant.

**Figure 8:**
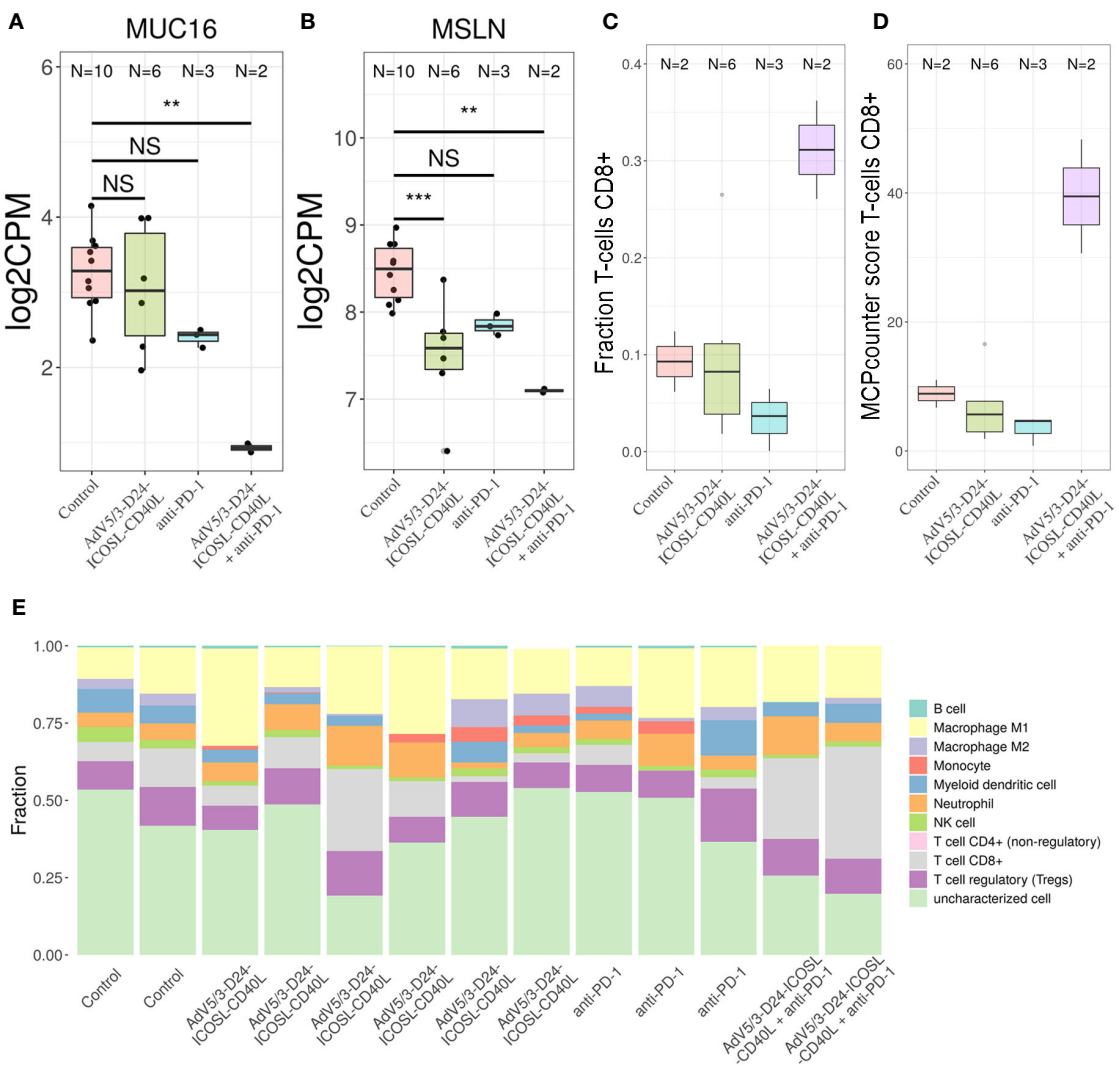
Transcriptomic analyses. To determine the putative levels of different immune cells in our data, we used two methods—MCP counter33 and quanTIseq. Both methods decompose bulk RNA-seq expression matrix using expression profile of genes characteristic only to specific cell types. For MCP counter, each cell type is assigned with a score, which correlates with putative number of each cell line. For quanTIseq for each sample, a fraction of each of predefined cell types is provided. We found out that only the combinatorial therapy resulted in the increased levels of CD8+ T cells in TME. Transcriptomic analyses revealed that **(A)** MUC16 and **(B)** MSLN were found among genes that expression was substantially decreased by the combinatorial therapy; **p ≤ 0.05, ***p<0.01. Putative levels of CD8+ T cells in TME analyzed with- quanTIseq **(C)** and MCP counter **(D)** using bulk RNA-seq data. The combinatorial therapy resulted in the highest levels of CD8+ T cells. **(E)** The application of AdV5/3-D24-ICOSL-CD40L or pembrolizumab alone did not yield increased levels of this subpopulation of T cells. Composition of TME predicted with quanTIseq using bulk RNA-seq data. ns, not significant.

To better characterize the effect of the individual (anti-PD-1, AdV5/3-D24-ICOSL-CD40L) and combinatorial (AdV5/3-D24-ICOSL-CD40L + anti-PD-1) therapies, we performed gene expression analysis by RNA-seq on extracted human H226 mesothelioma xenografts explanted from the humanized mice. To increase statistical power of this analysis, we also included, as control, eight samples from publicly available RNA-seq data for the H226 cell line from the RNA Atlas (GSM4117346). The DGE analysis revealed 3,190, 1,158, and 825 differentially expressed genes compared to control (fold change in log2 scale at least 1, average expression at least −2, and adjusted p-value < 0.05) for AdV5/3-D24-ICOSL-CD40L, anti-PD-1, and AdV5/3-D24-ICOSL-CD40L+ anti-PD-1 therapies, respectively. A set of common 258 genes with expression altered in all therapies with respect to control was observed. A total of 241 genes were overexpressed, and 17 were underexpressed (**Supplementary Figure S4**). Of the 825 genes, 334 were unique to the combinatorial regimen. This group comprised of several genes associated with activated immune cells like CD83 (log fold change 1.38; adjusted p-value, 0.01, **Figure 7A**), CRTAM (2.22; 0.01, **Figure 7B**), CD8B (1,9; 0.03, **Figure 7C**), or CXCL9 (l2.08, 0.07, **Figure 7D**). Moreover, several more genes associated with T-cell activation were upregulated in samples treated with either AdV5/3-D24-ICSOL-CD40L or AdV5/3-D24-ICSOL-CD40L + anti-PD-1. These include chemokines CXCL3, CXCL10, CXCL11, and TAP1 (**Figures 7E–H**). For CXCL11, the observed expression increased by 2.71 and 1.14 for combinatorial and AdV5/3-D24-ICSOL-CD40L therapies, respectively. In samples treated exclusively with CPI, the expression of this protein was increased by 0.23. A similar pattern was observed for all the other above-mentioned genes. MUC16 (also known as CA125) was found among underexpressed genes that were mostly affected by the combinatorial therapy (fold change decreased by 2.33, adjusted p-value < 0.01). We have also observed decreased expression of MSLN in response to all types of treatments; however, the combinatorial therapy had the strongest effect on mesothelin levels (fold change decreased by 1.35; adjusted p-value, 0.01) (**Figure 8**). Levels of this proteins were not significantly altered in two remaining treatment strategies.

Gene Ontology analysis confirms the abovementioned conclusions, as the genes belonging to the “adaptive immune response” GO category are overrepresented among genes with altered expression in the combinatorial regimen compared to control (p-value 5e−5, **Supplementary Figure S5**). Other enriched GO category includes “cell surface receptor signalling pathway,” “response to wounding,” and “response to estrogen.” Finally, we decided to check a putative composition of TME observed with our bulk RNA-seq data. Several studies point out that the tumor material analyzed with RNA-sequencing is often contaminated with non-tumor cells, for example tumor-infiltrating immune cells (30), like cytotoxic CD8+ T cells. These cells are able to recognize and eradicate tumor cells and are associated with a good clinical prognosis in different cancer types and have an instrumental role in an anti-PD-1 immunotherapy. To determine the putative levels of different immune cells in our data, we used two methods—MCP counter (33) and quanTIseq (30). Both methods decompose bulk RNA-seq expression matrix using expression profile of genes characteristic only to specific cell types. For MCP counter, each cell type is assigned with a score, which correlates with putative number of each cell line. For the quanTIseq for each sample, a fraction of each of predefined cell types is provided. We found out that only the combinatorial therapy resulted in the increased levels of CD8+ T cells in TME (**Figure 8**). The application of AdV5/3-D24-ICOSL-CD40L or pembrolizumab alone did not yield increased levels of this subpopulation of T cells. Gene expression was confirmed by qPCR analyses.

## Discussion

Despite huge efforts to improve the understanding and treatment of malignant mesothelioma, clinical practice has not changed dramatically in recent decades. To accelerate the development of novel treatment options, rational and well-designed investigations should be performed, and personalized approaches should be investigated (3, 34). In conjunction with CPIs and chemotherapy, OVs have shown to have a synergistic anti-cancer impact (1, 14, 25). While OVs exhibit clinical potential and a safety attribute in existing immunogenic tumors, clinical response rates are mild (35). As a result, the efficiency of oncolytic vectors must be improved in an attempt to implement them as a universal cancer treatment option.

In such a context, we designed the AdV5/3-D24-ICOSL-CD40L (25) oncolytic vector and tested its anti-cancer effectiveness along with an anti-PD-1 antibody in both immunodeficient and humanized xenografted mesothelioma H226 mouse model. The oncolytic adenovirus AdV5/3-D24-ICOSL-CD40L showed comparable anti-cancer efficacy to the treatment with AdV5/3-D24 in tested mesothelioma cell lines *in vitro*. The observed results are in corroboration with the ones reported elsewhere (14, 25, 36). Results suggest that incorporation of ICOSL and CD40L expression cassette into the genome of AdV5/3-D24-ICOSL-CD40L did not impair oncolytic properties of the vector when compared to AdV5/3-D24.

When contrasted to the other treatment groups, cell line treated with AdV5/3-D24-ICOSL-CD40L in conjunction with anti-PD-1 demonstrated significantly higher levels of calreticulin exposure and ATP. All these are known hallmarks of activated immunogenic cell death pathway (37). CD91, P2RX7, and TLR4 on dendritic cells are the receptors specific for CRT, ATP, and HMGB1, respectively. The ATP-P2RX7 signaling pathway attracts DCs to the target tumor tissue, the CRT-CD91 pathway enhances DC engulfment of cancer antigens, and the HMGB1-TLR4 route enables the optimum display of cancer antigens (38). Consequently, DC antigen uptake and presentation are thereby improved, resulting in a more adaptable antitumor immune response (39). These findings could indicate that the combinatorial therapy conferred a more cytotoxic immunological effect than the other groups.

Based on the promising *in vitro* results showcased by the combinatorial therapy involving AdV5/3-D24-ICOSL-CD40L, AdV5/3-D24 and their combinations with anti-PD-1, *in vivo* experiments were conducted. The animal model was built on H226 cells, where the virus showed the most effective oncolytic properties *in vitro*. The anti-tumor efficacy of oncolytic adenoviruses (AdV5/3-D24-ICOSL-CD40L or AdV5/3-D24) in mesothelioma H226 xenograft BALB/c nude immunodeficient model illustrated superior reduction in tumor volume when compared to control. Anti-cancer effectiveness was noted in mice treated with oncolytic adenovirus and combination therapy, although no effect of anti-PD-1 was observed. Nevertheless, it is important to underline that the model based on nude BALB/c mice is immunodeficient, and the observed anti-tumor effects have not included any contribution from the hosts’ adaptive immune responses. The treatment regimens were well tolerated, and necropsy evaluation revealed no pathological alterations.

Tested oncolytic adenovirus AdV5/3-D24-ICOSL-CD40L is a human-specific vector, replicating selectively in human cancer cells. Anti-PD-1 inhibitor (pembrolizumab) is also a human-specific drug. Therefore, considering the features of the proposed biological agents a proper animal model is a crucial prerequisite for further investigation. To this point, humanized mouse model was utilized to test anti-cancer properties, allowing to investigate both oncolytic properties of the vector and immune responses in human immune system. In fact, improved anti-cancer efficacy was observed in humanized xenograft H226 mesothelioma NSG mouse model [improved anti-cancer efficacy was observed in combination therapy with AdV5/3-D24-ICOSL-CD40L plus anti-PD-1 versus mock (p ≤ 0.01) and in mice treated with AdV5/3-D24-ICOSL-CD40L (vs. mock, p ≤ 0.05)] implying the suitable entry of adenovirus in the tumor cells, which can further lead to better anti-cancer effectiveness of the proposed therapy. The said fact was evidenced by the expression of CAR, DSG-2, and CD46 receptors and PD-L1 in human mesothelioma cell lines.

Our results corroborate with the outcome of a study utilizing the Ad5/3-D24-GM-CSF virus with pembrolizumab in the humanized A2058 melanoma huNOG mouse model (14, 21). In that study, the authors observed a drop in tumor volume as compared to pembrolizumab monotherapy (14, 21). In fact, therapy with another oncolytic virus T-VEC with pembrolizumab was also well tolerated, with no associated toxicities, and a spike in intra-tumoral CD8+ T cells, raised PD-L1 expression, and IFN- gene expression was observed, according to the clinical study reports (Phase Ib) (40). These findings suggest that through altering the TME, oncolytic vectors could improve pembrolizumab efficacy. These superior efficacy results lend up a scope in extending overall survival in patients with melanoma. In line with these findings, Cook et al. (41) showed that Coxsackievirus A21 (CAVATAK) synergized in anti-cancer efficacy when administered with immune CPIs. A trial assessing CAVATAK with ipilimumab resulted in 50% objective responses in melanoma patients (18, 41). Encouraging data have been also reported in a treatment with VALO-D102, an oncolytic vector, encoding for OX40L and CD40L, used in PeptiCRAd cancer vaccine system. The local administration of PeptiCRAd strongly elevated tumor-specific T-cell responses, inhibited tumor growth, and in combination with anti-PD-1, significantly improved anti-cancer effect (18, 23). Similar data have been also reported by Hemminki lab where i.t. administration of oncolytic adenovirus expressing TNFa and IL-2 improved systemic response to anti-PD-1 therapy, by re-shaping the TME of both injected and non-injected tumors (22), further supporting the rationale for combination of anti-PD-1 with oncolytic vectors virus for the treatment of human cancer.

Adenoviral DNA copy number as assessed by qPCR revealed viral DNA presence in tumor cells. This is not a surprise, as adenoviral vectors target tumors by multiplying in and destroying cancer cells resulting in tumor death. The afflicted tumor cell experiences lysis when the replication cycle is completed, releasing offspring virions that are competent enough for infecting neighboring tumor cells and successive rounds of vector replication and cell lysis thereby kill the tumor (42). Exogenous proteins expressed by certain genes that affect anti-cancer action or their expression can be manipulated to prevent virus multiplication in cancer cells (43). To this point, *ex vivo* analysis indicated local synthesis of ICOSL and CD40L transgenes encoded by the AdV5/3-D24-ICOSL-CD40L. In solid tumors, ICOSL expression promotes the stimulation of CD8+ cytotoxic T cells, resulting in anti-tumor immune function. In turn, CD40L triggers maturation of antigen presenting cells (APCs). Surprisingly, ICOSL transfected tumor cells indicated that the ligand promotes tumor regression by activating CD8+ cytotoxic T-mediated mechanisms (44). We can speculate that the presence of both co-stimulatory molecules, mamely, CD40L and ICOSL, encoded by the vector contributed to enhanced infiltration of GrB+ CD8+ T cells in the tumors.

This could be the cause for the combo therapy’s better *in vivo* efficacy when compared to other treatment regimens. Importantly, antineoplastic efficacy was connected to reduced tumor volume and enhanced infiltration of TILs, including activated cytotoxic T cells (GrB+ CD8+). Moreover, we have observed a negative correlation between tumor volume and GrB+CD8+ TILs, confirming the importance of anti-cancer immune responses in cancer growth control. Indeed, current findings indicate that the presence of CD8+ T cells is predictive of anti-PD-1 therapeutic responses in malignancies such as non-small-cell lung carcinoma and melanoma (43). Therefore, enhancing the infiltration of activated cytotoxic (GrB+CD8+ T cells) tumor-infiltrating T cells by the combinatorial therapy, we can induce anti-cancer effect.

Transcriptomic analysis revealed upregulation of various genes, such as, CD83, CRTAM, CXCL11, and TAP1 only in samples treated with combinatorial therapy. In fact, CD83 is found on a variety of activated immune cells, although it is stably expressed by mature dendritic cells (DC). CD83 also regulates maturation, activation, and homeostasis. Interaction between T cells and APCs is required for optimal TCR activation and development (45) of anti-cancer immune responses. It has been demonstrated that one of gene upregulated during T-cell activation is CRTAM, on both human CD4+ and CD8+ T cells (45). This observation is consistent with previous reports that human CRTAM transcripts are transiently detected in activated CD8+ T cells and NK and NKT cells (45). Levels of CXCL11 correlates with antitumor immunity and an improved prognosis in colon cancer (46). Kristner et al. also showed that the expression of CXCL11 allowed the most stringent prediction of overall survival and disease-free survival in colon colorectal cancer (47), suggesting anti-cancer role of CXCL11. TAP1 is an ABC transporter that forms a TAP complex together with TAP2, levels of which remain relatively stable between analyzed conditions. The transporter TAP allows peptides to enter the ER that can subsequently bind to MHC I molecules for presentation to CD8+ T cells (48). Gene set enrichment analysis indicates that genes with altered expression in combinatorial therapy belong to the “cell surface receptor signaling pathway” and “adaptive immune response” GO category. Decomposition of bulk RNA-seq data also indicate that the increased levels of CD8+ T cells are only observed in TME after the combinatorial therapy. All this supports a notion of enhanced T-cell activation in TME following introduction of CPI together with the modified adenovirus.

Transcriptomic analysis indicates that the combinatorial therapy has the strongest effect on lowering mesothelin and MUC16 levels (confirmed by gene expression analyses). To date, mesothelin is the only tumor biomarker to receive US FDA approval for clinical use in mesothelioma. Mesothelin is usually expressed on the surface of mesothelial cells, and in the cancerous phase, it can be present in the blood (49). Importantly, increased survival rate of patients with ovarian cancer was observed for the group with lowered levels of this protein (50). Our data suggest that lowered mesothelin expression level correlates with anti-cancer efficacy observed in animal studies, thus strengthening the rationale for combinatory treatment using oncolytic adenovirus AdV5/3-D24-ICOSL-CD40L with anti-PD-1 in mesothelioma therapy.

## Conclusions

Our *in vivo* studies confer the fact that oncolytic viruses expressing powerful immune modulators could be used to boost the systemic potency of immune CPIs. Therefore, our preclinical observations endorse the concept that by specifically targeting cancer cells and inducing immunogenic cell death, the proposed combinatorial therapy could enhance the anti-cancer performance and the overall survival. Nevertheless, further studies need to be conducted to confirm reported findings.

## Data availability statement

The transcriptomic data has been deposited at National Library of Medicine (Accession: PRJNA1010481).

## Ethics statement

Ethical approval was not required for the studies on humans in accordance with the local legislation and institutional requirements because only commercially available established cell lines were used. The animal study was approved by Animal procedures were approved by the Austrian Federal Ministry of Science and Research, the Italian Ministry of Health, and the Warsaw University of Life Sciences’ II Local Ethical Committee for Animal Experiments. The study was conducted in accordance with the local legislation and institutional requirements.

## Author contributions

MG: Data curation, Investigation, Methodology, Resources, Writing – original draft, Writing – review & editing. MW: Resources, Writing – original draft, Writing – review & editing. IA: Data curation, Investigation, Resources, Writing – review & editing. MS: Investigation, Resources, Writing – review & editing, Writing – original draft. ML: Resources, Writing – review & editing, Formal Analysis, Methodology, Software, Writing – original draft. MP: Resources, Writing – review & editing. AZ: Resources, Writing – review & editing. TS: Resources, Writing – review & editing, Data curation, Formal Analysis, Software. DP: Formal Analysis, Resources, Software, Writing – review & editing. SS: Resources, Writing – review & editing. PC: Resources, Writing – review & editing. VC: Resources, Writing – review & editing. RA: Resources, Writing – review & editing. BR: Resources, Writing – review & editing, Data curation. KP: Resources, Writing – review & editing, Writing – original draft. LK: Conceptualization, Data curation, Formal Analysis, Funding acquisition, Investigation, Methodology, Project administration, Resources, Software, Supervision, Visualization, Writing – original draft, Writing – review & editing.
