# Supplementary material for: Novel combinatorial therapy of oncolytic adenovirus AdV5/3-D24-ICOSL-CD40L with anti PD-1 exhibits enhanced anti-cancer efficacy through promotion of intratumoral T-cell infiltration and modulation of tumour microenvironment in mesothelioma mouse model
Source: Front Oncol. 2023 Nov 20;13:1259314. doi: 10.3389/fonc.2023.1259314 (PMC10694471; doi:10.3389/fonc.2023.1259314)
Supplement: Supplementary file 1 [file DataSheet_1.pdf]

Table S1. Characteristics of clinical signs in animal health scoring.

Clinical sign	Abb.	Description	Score
**Coat**	C	Normal<br>Lack of grooming, partial alopecia<br>Massive alopecia, wounds, bleedings, inflammation	0<br>1<br>2
**Movement**	M	Normal<br>Slow movements, paralysis of one member<br>Difficulties to eat or drink, paralysis of more than one member	0<br>2<br>3
**Activity**	A	Normal<br>Agitates, over-reactive hypo-reactive<br>Prostrated	0<br>1<br>3
**Paleness**	P	Normal<br>Slight<br>Severe	0<br>1<br>2
**Movement**	M	Normal<br>Segmentation of the vertebral column evident, pelvic bones palpable<br>Emaciated, skeletal structure prominent	0<br>2<br>3

Table S2. Treatment characteristics in immunodeficient xenograft H226 mesothelioma mouse model (BALB/c nude, n= 5 mice per group; 5 tumors per group). Mice were treated on Days 0, 3, 6, 9, 12 and 15 post tumor formation.

Group	Day 0	Day 3	Day 6	Day 9	Day 12	Day 15
**1. Control**	PBS	PBS	PBS	PBS	PBS	PBS
**2. AdV5/3-D24**	$1x10^8$ VP i.t.	$1x10^8$ VP i.t.	$1x10^8$ VP i.t.	$1x10^8$ VP i.t.	$1x10^8$ VP i.t.	$1x10^8$ VP i.t.
**3. AdV5/3-D24-ICOSL-CD40L**	$1x10^8$ VP i.t.	$1x10^8$ VP i.t.	$1x10^8$ VP i.t.	$1x10^8$ VP i.t.	$1x10^8$ VP i.t.	$1x10^8$ VP i.t.
**4. Anti PD-1**	200 µg i.v.	200 µg i.v.	200 µg i.v.	200 µg i.v.	200 µg i.v.	200 µg i.v.
**5. AdV5/3-D24 + anti PD-1**	$1x10^8$ VP i.t. + 200 µg i.v.	$1x10^8$ VP i.t. + 200 µg i.v.	$1x10^8$ VP i.t. + 200 µg i.v.	$1x10^8$ VP i.t. + 200 µg i.v.	$1x10^8$ VP i.t. + 200 µg i.v.	$1x10^8$ VP i.t. + 200 µg i.v.

**6. AdV5/3-D24-ICOSL-CD40L + anti PD-1**	$1x10^8$ VP i.t. + 200 µg i.v.	$1x10^8$ VP i.t. + 200 µg i.v.	$1x10^8$ VP i.t. + 200 µg i.v.	$1x10^8$ VP i.t. + 200 µg i.v.	$1x10^8$ VP i.t. + 200 µg i.v.	$1x10^8$ VP i.t. + 200 µg i.v.

Table S3. Treatment characteristics in humanized xenograft H226 mesothelioma mouse model (NSG mouse, n=4 mice per group; 8 tumors per group). Mice were treated on Days 0, 3, 6, 9, 12 and 15 post tumor formation. The dose/mouse is indicated in the table.

**Group**	**Day 0**	**Day 3**	**Day 6**	**Day 9**	**Day 12**	**Day 15**
**1. Control**	PBS	PBS	PBS	PBS	PBS	PBS
**2. AdV5/3-D24-ICOSL-CD40L**	$2x10^9$ VP i.t.	$2x10^9$ VP i.t.	$2x10^9$ VP i.t.	$2x10^9$ VP i.t.	$2x10^9$ VP i.t.	$2x10^9$ VP i.t.
**3. Anti PD-1**	200 µg i.v.	200 µg i.v.	200 µg i.v.	200 µg i.v.	200 µg i.v.	200 µg i.v.
**4. AdV5/3-D24-ICOSL-CD40L + anti PD-1**	$2x10^9$ VP i.t. + 200 µg i.v.	$2x10^9$ VP i.t. + 200 µg i.v.	$2x10^9$ VP i.t. + 200 µg i.v.	$2x10^9$ VP i.t. + 200 µg i.v.	$2x10^9$ VP i.t. + 200 µg i.v.	$2x10^9$ VP i.t. + 200 µg i.v.

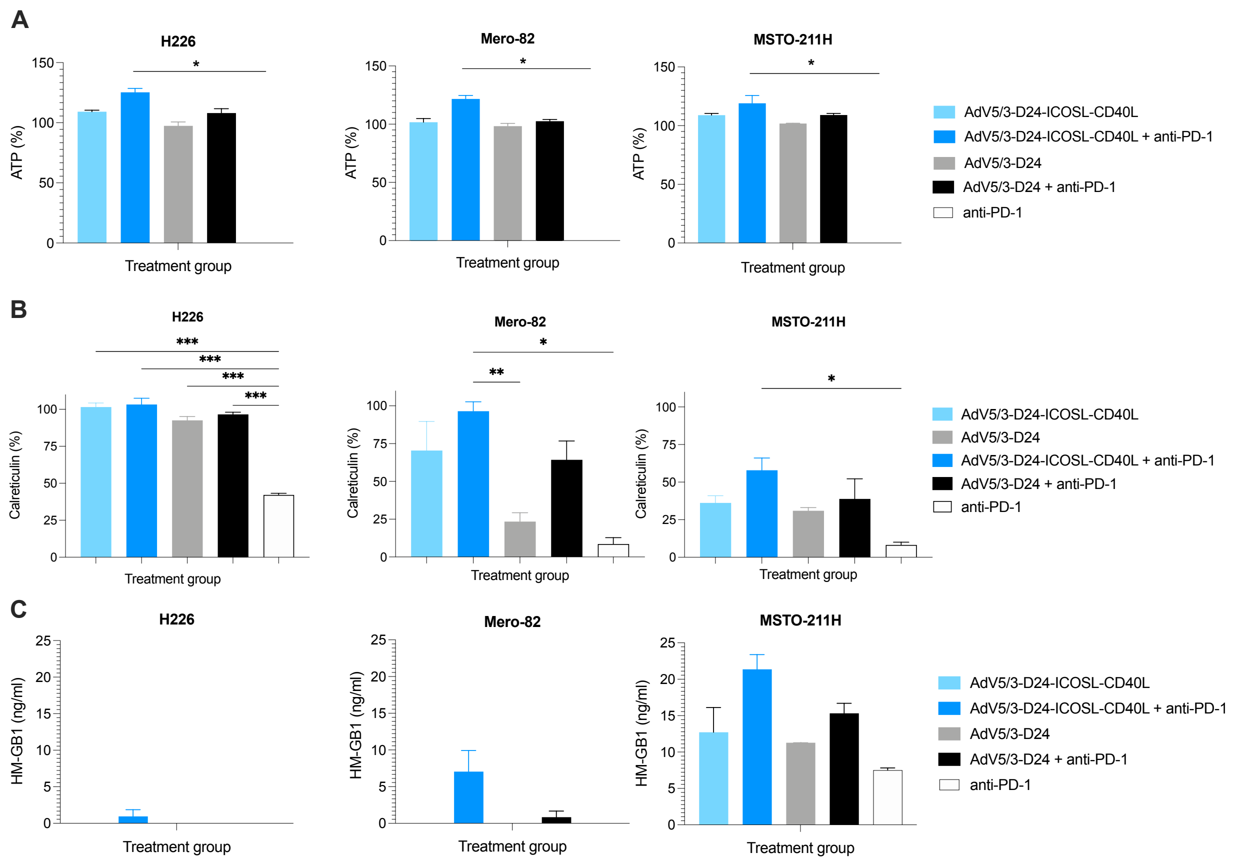

Figure S1. Evaluation of Immunogenic cell death. **A** Assessment of ATP release after the treatment (the control absorbance value was set to 100%). **B** CRT exposure in mesothelioma cell lines after treatment with oncolytic adenoviruses AdV5/3-D24-ICOSL-CD40L and AdV5/3-D24, as well as anti PD-1 therapy. Anti-calreticulin antibody staining with AlexaFluor® C was used to quantify CRT exposure (1x10^4 events were analysed for each sample, event count for untreated cells were set to correspond to 100% and the percentage of treated cells was expressed as a percentage of the untreated control event value for calreticulin measurements). **C** Evaluation of HMGB-1 release following treatment with oncolytic adenoviruses and anti PD-1 therapy. The HMGB-1 level was determined using an ELISA kit from the supernatant collected 72 hours after infection. Statistical analyses were carried out with one-way ANOVA. Error bars, mean±SEM, *, $p \leq 0.05$; **, $p \leq 0.01$; ***, $p < 0.001$.

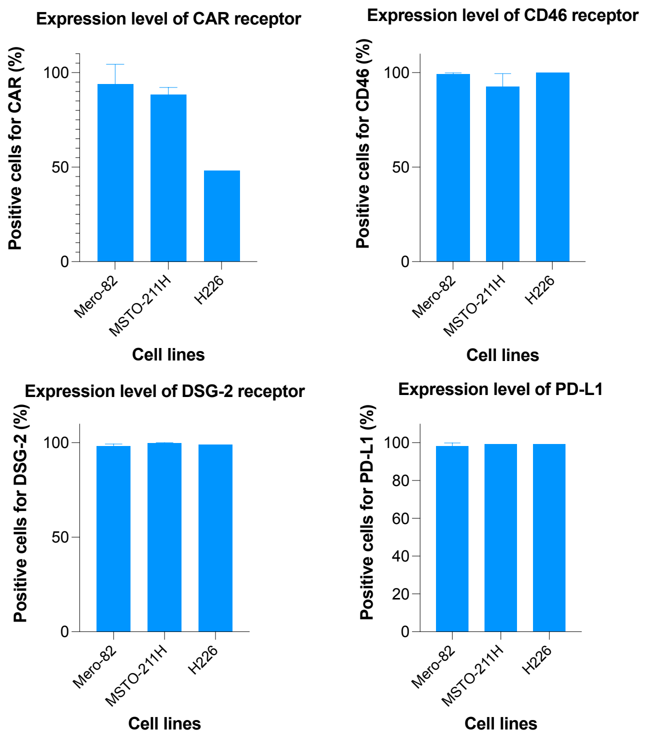

Figure S2. Expression of Coxsackie-Adenovirus Receptor (CAR) and Desmoglein-2 (DSG-2), CD46 receptors and PD-L1 in human mesothelioma cell lines, measured with flow cytometry with BD FACSCantoTM II (Franklin Lakes, NJ, USA). Data are expressed as percentage of cell positive for the marker. Error bars, mean±SEM.

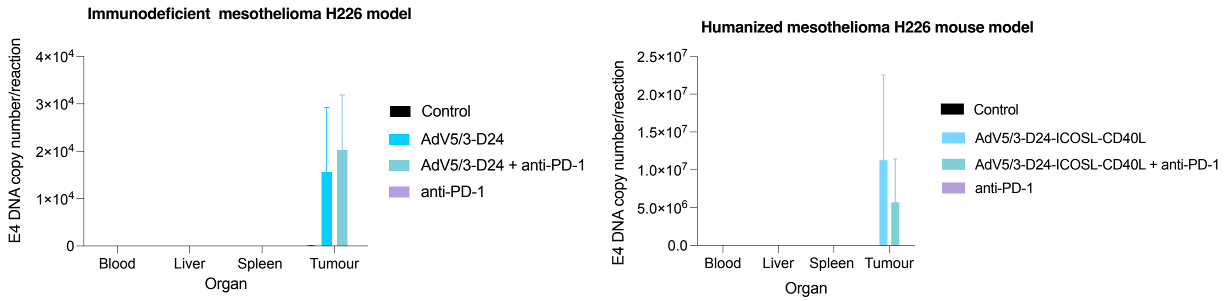

Figure S3. **Adenoviral copies towards E4 gene were measured by qPCR from euthanized mice's organs at the end of the treatment** (from immunodeficient mesothelioma H226 BALB/c nude (AdV5/3-D24) mice and humanized H226 mice (AdV5/3-D24-ICOSL-CD40L). Statistical analyses were carried out with ANOVA test. Error bars, mean±SEM.

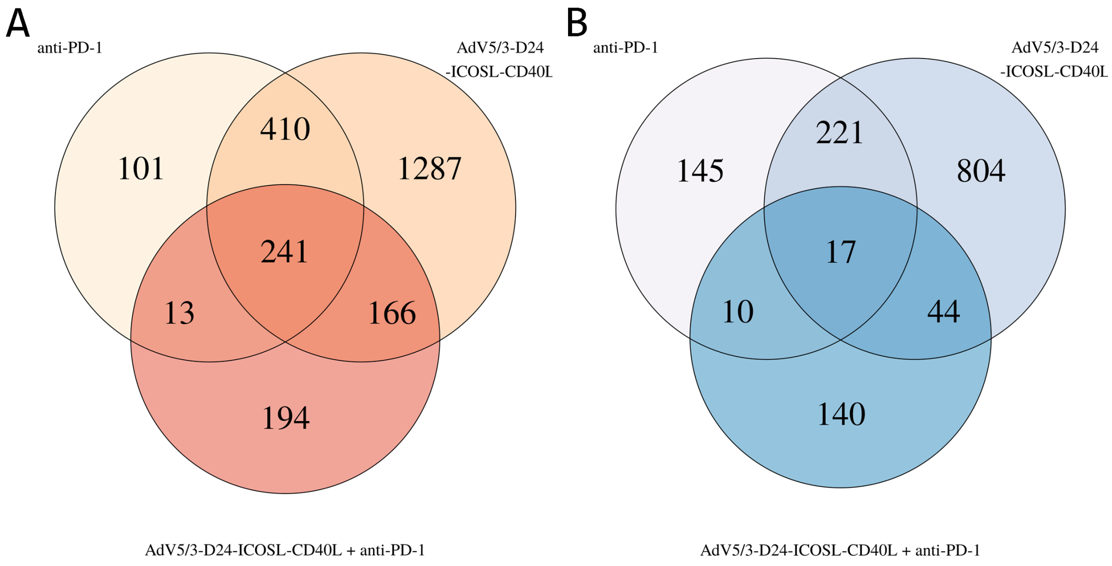

Figure S4. Venn plots indicating number of genes over-expressed **A** or under-expressed **B** in tested conditions with respect to control. A total of 258 genes was altered in all three treatment regimens (241 genes over- and 17 under-expressed). For combinatorial therapy, 334 out of 825 genes were unique to this condition.

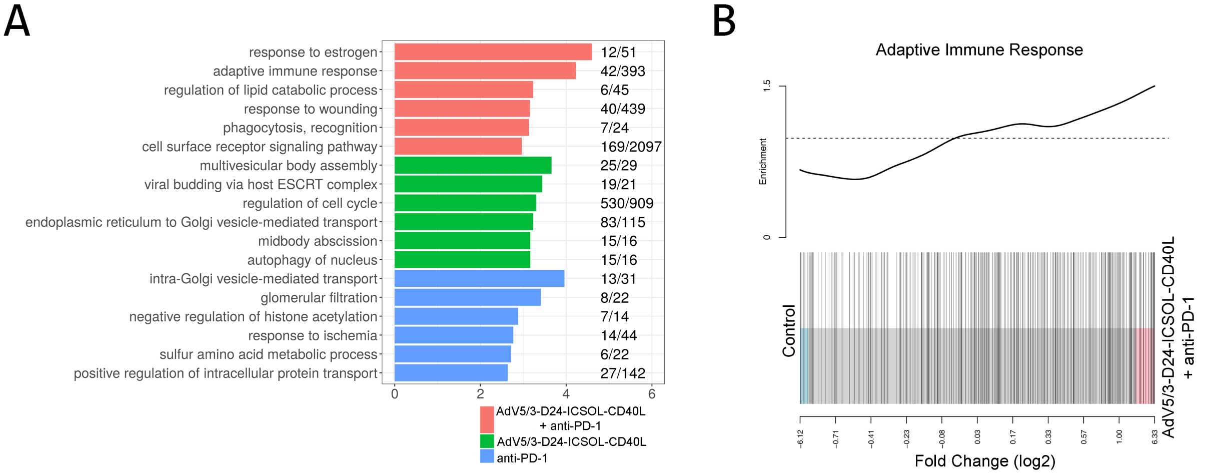

Figure S5. Gene Ontology analysis on the genes affected by different types of therapies. **A** Top 6 categories for each group. Genes affected by combinatorial therapy showed enrichment of "adaptive immune response", ''phagocytoses'', "cell surface receptor signalling pathway'', "response to wounding'' categories. **B** GSE plot for adaptive immune responses, indicates a general increase in expression of genes belonging to that category.
